# Comparison of In Vitro Approaches to Assess the Antibacterial Effects of Nanomaterials

**DOI:** 10.3390/jfb13040255

**Published:** 2022-11-19

**Authors:** Abdulkader Masri, David M. Brown, David G. E. Smith, Vicki Stone, Helinor J. Johnston

**Affiliations:** School of Engineering and Physical Sciences, Institute of Biological Chemistry, Biophysics and Bioengineering, Heriot-Watt University, Edinburgh EH14 4AS, UK

**Keywords:** nanomaterials, antibacterial, in vitro, nanotoxicology, copper oxide

## Abstract

The antibacterial properties of nanomaterials (NMs) can be exploited in a range of consumer products (e.g., wound dressings, food packaging, textiles, medicines). There is also interest in the exploitation of NMs as treatments for infectious diseases to help combat antibiotic resistance. Whilst the antibacterial activity of NMs has been assessed in vitro and in vivo in numerous studies, the methodology used is very varied. Indeed, while numerous approaches are available to assess the antibacterial effect of NMs in vitro, they have not yet been systematically assessed for their suitability and sensitivity for testing NMs. It is therefore timely to consider what assays should be prioritised to screen the antibacterial properties of NMs. The majority of existing in vitro studies have focused on investigating the antibacterial effects exhibited by silver (Ag) NMs and have employed a limited range of assays. We therefore compared the antibacterial effects of copper oxide (CuO) NMs to *Escherichia coli*, *Pseudomonas aeruginosa*, *Staphylococcus aureus*, and *Bacillus subtilis* at various concentrations (12.5–200 µg/mL) using a battery of tests (well and disc diffusion, plate counts—time-kill method, optical density measurement—OD, Alamar Blue and live/dead viability assays, and quantitative polymerase chain reaction). CuO NMs were most toxic to *B. subtilis* and *E. coli*, while *P. aeruginosa* was the least sensitive strain. All assays employed detected the antibacterial activity of CuO NMs; however, they varied in their sensitivity, time, cost, technical difficulty and requirement for specialized equipment. In the future, we suggest that a combination of approaches is used to provide a robust assessment of the antibacterial activity of NMs. In particular, we recommend that the time-kill and OD assays are prioritised due to their greater sensitivity. We also suggest that standard operating protocols are developed so that the antibacterial activity of NMs can be assessed using a harmonised approach.

## 1. Introduction

Nanomaterials (NMs) are generally considered to have at least one dimension that is <100 nm in diameter. New properties (such as greater strength, stability, electrical and thermal conductivity, and biological activity) emerge in materials at the nanoscale [1]. Therefore, the exploitation of NMs is increasing across a range of applications in many industrial sectors. It is established that some metal/metal oxide NMs—e.g., silver (Ag), copper oxide (CuO) and zinc oxide (ZnO) and titanium dioxide (TiO_2_)—have antibacterial effects which is driving their exploitation in a range of products and biomedical applications (e.g., food packaging, textiles, wound dressings, cosmetics and personal care products) [2]. NMs may also be used as alternatives to antibiotics to treat infectious diseases and help combat antibiotic resistance [3].

However, whilst many studies have been performed to assess the antibacterial properties of NMs that can be harnessed in different applications, the antibacterial effects of NMs could also lead to harmful effects on human health. For example, NMs with antibacterial activity may be intentionally or accidentally ingested, and lead to a disturbance in the gut microbiota that could lead to adverse health impacts as observed following oral exposure to some antibiotics [4]. Indeed, there is already evidence that NMs can influence the composition of the gut microbiota. For instance, Ag NMs changed the composition of gut microbiome in rats so that a larger proportion of Gram-negative bacteria was observed after oral administration for 13 weeks [5]. Moreover, SiO_2,_Ag and TiO_2_NMs showed a detrimental impact on the diversity and composition of the gut microbiome in mice after oral administration at doses relevant to human dietary exposure [4]. These effects are not limited to rodent species and a recent study showed that CuO NMs disturbed the structure and diversity of the gut microbiome of the invertebrate *Folsomia candida* following exposure via soil [6]. Furthermore, Cu and Ag NMs changed the composition of the microbial population present in the intestine of zebrafish following dietary intake [7]. However, not all impacts of (antibacterial) NMs following oral exposure can be considered adverse. For example, it has been demonstrated that Cu complexed to chitosan NMs targeted pathogenic bacteria such as *Salmonella*, while sparing beneficial intestinal commensal strains such as *Lactobacilli* [8]. Assessment of the antibacterial properties of NMs is therefore required to assess their potential beneficial and detrimental impacts on human health.

A number of in vitro methods are available to assess the antimicrobial effects of substances, including NMs. One of the most frequently performed approaches for determining the bactericidal effect of NMs against both planktonic and biofilm-growing bacteria is the time-kill assay [9,10]. This method assesses a time- and/or a concentration-dependent antibacterial effect using agar plate colony counting. Significant bactericidal activity is defined by the Clinical and Laboratory Standards Institute (CLSI) as a reduction in the number of colonies that grow on an agar plate over time by ≥3 log_10_ colony forming units (CFU)/mL compared to the initial inoculum, whereas bacteriostatic activity corresponds to <3 log_10_CFU/mL [11,12,13]. CuO NMs were shown to be efficient at killing *E. coli*, *S. aureus*, *P. aeruginosa*, Methicillin resistant *S. aureus* (MRSA), and *Proteus* spp. in vitro, when the time-kill assay was used [14,15]. The choice of medium used in the time-kill method can influence the results when assessing NM toxicity. For example, the presence of proteins, salts, and glucose increased diamond NM aggregation resulting in a decrease in their antibacterial activity [16]. Phosphate-buffered saline (PBS—which lacks nutrients for bacterial growth, but has the ability to maintain pH and provides a stable, inert environment for the bacteria) has therefore been used in the time-kill assay in some studies instead of a nutrient-rich broth to assess the antibacterial activity of NMs [16,17]. Antibacterial effects can also be detected by monitoring optical density (OD) by spectrophotometry over time to determine the growth of bacteria in a suspension. Numerous studies have assessed the antibacterial efficacy of NMs by applying the OD method [18,19]. Agar well and disc diffusion assays have also been exploited for the evaluation of antibacterial effects of NMs based on the size of the inhibitory zone [20,21]. For example, the well diffusion assay showed that Au NMs displayed strong antibacterial activity against Gram-negative bacteria (*E. coli*, *P. aeruginosa*, *S. typhimurium*) and moderate activity against Gram-positive strains (*B. subtilis*, *S. aureus*, *S. pyogenes*) [22]. Moreover, Ag NMs showed notable bactericidal activity against *L. monocytogenes* and *S. enterica* using this approach [23]. Furthermore, Kim et al. (2007) used the disc diffusion method to report greater biocidal efficacy of Ag NMs for *E. coli* when compared to the mild inhibitory effect observed for *S. aureus*, which was attributed in part to differences in cell structure between Gram-positive and Gram-negative bacteria [24]. Viability assays can be employed to assess the antibacterial properties of NMs such as the Alamar Blue and live/dead assays. For the Alamar Blue assay, only metabolically active, viable bacterial cells reduce the non-fluorescent (blue) resazurin dye to a fluorescent (red) product (resorufin) and so cell viability can be monitored via assessment of fluorescent intensity [25]. The live/dead assay uses fluorescent dyes to assess cell viability; SYTO 9 penetrates the membrane of viable bacterial cells to stain them green, while propidium iodide (PI) is excluded from viable cells with intact membranes and stains dead cells red [26]. To date, however, these assays have not been widely used to assess the antibacterial properties of NMs.

Quantitative polymerase chain reaction (qPCR) is also now being more widely used to evaluate the efficacy of antibacterial compounds [27,28]. SYBR green is a fluorescent stain which binds to double-stranded DNA with the strength of the fluorescent signal indicating the amount of DNA amplicons in the sample at a specific time (i.e., if there are fewer bacterial cells, there is a lower fluorescent signal) [29]. Furthermore, PCR can be used to distinguish between live and dead bacteria by using propidium monoazide (PMA). Metabolically active cells with an intact membrane exclude PMA and are therefore considered viable. However, if membrane integrity is compromised, PMA penetrates the cell and interacts with DNA in dead cells. The labelled DNA is then not available for the amplification by qPCR, and the difference of Threshold Cycle (CT value) between untreated and treated cells indicates what proportion of treated cells are viable [28,30]. A summary of the benefits and limitations of each in vitro antibacterial assay is presented in Table 1. 

In antibacterial testing, the experimental outcome is impacted by many factors, such as the NM dispersion protocol used, the bacteria species/strain selected, the inoculum preparation used, the type of culture medium used, the incubation conditions (e.g., temperature, humidity, aeration and shaking) and the approach used to assess their antibacterial activity [10]. For example, there is evidence that the composition of exposure media (pH, ionic strength, salt concentrations, quality and quantity of natural organic matter) and media type (solid vs. liquid) strongly influences the antibacterial activity of NMs in vitro [36,43]. A lack of standard protocols to assess the antibacterial properties of NMs makes comparison of the results from different studies difficult, and further, can lead to contradictory data between studies [27,36].

A large number of NMs require testing, and to improve reproducibility between studies, there is a need for standardised and efficient tests to assess their antibacterial activity. Existing studies investigating the antibacterial properties of NMs in vitro typically only use a limited number of assays, with plate counts and well/disc diffusion assays being the most commonly employed. The more widespread application of other assays (e.g., measurement of OD, viability assays, and PCR) could make testing more sensitive, rapid and with higher throughput. Accordingly, it is timely to consider the advantages and limitations of different approaches to inform which assays should be prioritised in the future when assessing the antibacterial properties of NMs. The aim of this study was therefore to compare the sensitivity of different approaches to assess the antibacterial activity of NMs in vitro, namely: bacterial growth over time through OD measurement (growth curve); diffusion methods (well and disc); time-kill assay (plate counts); viability assays (Alamar Blue and live/dead); and quantitative PCR.

The majority of existing studies in the published literature have focused on assessing the antibacterial properties of Ag NMs. CuO NMs were selected for investigation in our study as the antibacterial properties of CuO NMs can be exploited in wide ranging applications including antibacterials, food packaging, and textiles [44,45]. In addition, both in vitro and in vivo studies have already demonstrated that CuO NMs exhibit antibacterial properties [46,47,48]. For example, CuO NMs exhibited antibacterial activity against *S. aureus, E. coli*, and *P. aeruginosa* when assessed via disc and well diffusion assays, investigation of ATP concentration (viability assay) and OD measurements [49]. In addition, the time-kill assay has shown that CuO NMs exhibits antibacterial properties to *S. aureus, E. coli*, *P. aeruginosa*, and *S. Epidermidis* [50]. Furthermore, Azam et al. (2012a) demonstrated that CuO NM toxicity to *E. coli* and *P. aeruginosa, B. subtilis* and *S. aureus* was size-dependent using the well diffusion method [51]. Only a limited number of rodent studies have assessed whether ingested CuO NMs disrupt the intestinal microbiota [52]. Interestingly, the microbiome of earthworms was altered when exposed to CuO NMs [53]. However, Sizntsov et al. (2018) demonstrated that CuO NMs included in the feed did not alter probiotics or the major representatives of the microbial population in poultry [54]. Furthermore, the mechanism of NM toxicity has been probed in existing studies with the following identified as possible mechanisms by which NMs may cause their antibacterial effect: (1) ion release from CuO NMs, (2) interaction of NMs with the bacterial cell envelope to disrupt its normal structure and function [55], (3) penetration of NMs through openings in the bacterial membrane and subsequent binding to genetic structures, proteins, and organelles to promote cell death [56], and (4) stimulation of reactive oxygen species (ROS) production in bacterial cells [47]. For example, CuO NMs exhibited concentration dependent antibacterial activity against *E. coli*, *B. subtilis* and *S. aureus* due to release of Cu^2+^ ions [46]. In addition, *E. coli* and *S. aureus* treated with CuO NMs showed elevated cellular ROS levels which led to a reduction in adenosine triphosphate (ATP) production and therefore decreased bacteria viability [47]. Interestingly, existing studies have suggested that the physico-chemical properties of CuO NMs (e.g., size, solubility) may influence their antibacterial effects [24,51].

Gram-negative (*E. coli*, *P. aeruginosa*) and Gram-positive (*S. aureus*, *B. subtilis*) bacteria were selected for investigation in our study. *E. coli* are mostly commensal, although they also include several significant pathotypes which cause enteric and systemic diseases [57]. *P. aeruginosa* is widely present in the environment and may opportunistically cause serious infections, often in immunocompromised patients [58]. Although a major commensal of cutaneous and some mucosal surfaces, many strains of *S. aureus* are established to be resistant to various antibiotics (notably methicillin) and to be a well-known contaminant of medical equipment and health devices [59]. *B. subtilis* is found in the digestive tract of some animals and the soil and causes opportunistic infection and food poisoning [60,61]. In assessing antibacterial activities of NMs, the culture media, bacterial inoculum size and incubation conditions have been used as recommended by CLSI [10]. It is proposed that our findings can be used to help prioritise which assays are selected to assess the antibacterial properties of NMs in the future.

## 2. Materials and Methods

### 2.1. Physico-Chemical Properties of CuO NMs

The CuO NMs were obtained from PlasmaChem, Gmbh (Berlin, Germany). As reported by the manufacturer, these CuO NMs have a size of 15–20 nm, a specific surface area of 47 m^2^/g and a density of 6.3 g/cm^3^ [62]. CuO NMs have been characterized previously by Gosens et al. (2019) using several techniques [63]. Transmission Electron Microscopy (TEM) identified that the particle size of CuO NMs is 10 nm. Furthermore, X-ray diffraction (XRD) showed that CuO NMs had an average crystallite size of 9.3 nm [63]. Characterization of hydrodynamic diameter, zeta potential and PDI of a CuO NM suspension (50 µg/mL in sterile deionized water and bath sonicated for 16 min—see below) was performed using Dynamic Light Scattering (DLS, Malvern Zeta sizer Nano series).

### 2.2. Preparation of NMs

The stock suspension of NMs (1 mg/mL) was prepared in sterile deionized water and sonicated for 16 min in a bath sonicator (Max capacity 1.5 L. Ultrasonic power 36 W). The stock NM suspension was then diluted in fresh Mueller-Hinton broth (MHB) to concentrations of 12.5, 25, 50, 100, and 200 µg/mL, with the exception of the modified time-kill assay, in which the NMs were diluted in PBS.

### 2.3. Bacterial Strains

Stock cultures of *Escherichia coli* (NCTC12241), *Pseudomonas aeruginosa* (NCTC12903), *Staphylococcus aureus* (NCTC12973), and *Bacillus subtilis* (NCTC3610) were obtained from the National Collection of Type Cultures (NCTC). To ensure that a uniform number of bacteria were always used in each experiment, the number of colony forming units (CFU) for each strain was assessed by measuring turbidity at 600 nm using a UV/visible spectrophotometer (WPA, UK). The bacterial suspensions were diluted to a final concentration of approximately 5.0 × 10^5^ CFU/10 μL in MHB for all assays except the diffusion assays where the bacterial CFU was adjusted to a concentration of 1–2 × 10^8^ CFU/mL [10]. For all experiments, bacteria were incubated with CuO NMs at concentrations of 12.5, 25, 50, 100, and 200 µg/mL. MHB was included as a negative control and 100 µg/mL gentamicin as a positive control for all experiments.

### 2.4. Diffusion Assays (Well and Disc)

For both assays, 100 µL of each bacterial suspension was seeded using a sterile cotton spreader onto the surface of Mueller-Hinton agar (MHA) (<20 mL) in Petri dishes (100 mm D × 15 mm H). The plates were then left to dry at room temperature (RT) for 15–30 min.

For the well diffusion assay, four wells (6 mm in diameter) were made in each quarter of the MHA plates with a sterile steel-core borer (Figure S1). Well bases were sealed with one drop of molten agar. Wells were loaded with 50 µL of CuO NMs or the negative or positive control. Plates were left at RT for 1 h and then incubated at 37 °C for 18 h. The zone of inhibition around each well was measured in mm and well diameters (6 mm) were subtracted [51]. 

For the disc diffusion (Kirby-Bauer) assay, Whatman filter paper antibiotic discs (6 mm) were loaded with 10 µL of CuO NMs, negative or positive control, and the paper discs were then gently placed on the MHA plate. The plates were incubated for 18 h at 37 °C. The zone of inhibition around each paper disc was measured in mm and disc diameters (6 mm) were subtracted [31].

### 2.5. OD Measurement

Bacterial growth was assessed by measurement of OD over time in a 96 well plate. Briefly, 190 µL of CuO NMs, or the negative or positive control was added to each well and then 10 µL of each bacterial inoculum added. To evaluate NM interference, NMs in MHB alone (without bacteria) were included [64]. Plates were incubated at 37 °C for 24 h. Absorbance (optical density) was measured by using UV–Vis spectroscopy (microplate reader) (FLUOstar Omega, BMG LABTECH, Aylesbury, UK) at 600 nm every 2 h to generate a bacterial growth curve and to determine the LC_50_ concentration (concentration that causes a 50% reduction in the bacterial population) for CuO NMs [10,49,65]. OD values for each of the NM concentrations in the absence of bacteria were subtracted from the OD values measured in the presence of bacteria to account for possible NM interference with the OD readings.

### 2.6. Plate Count Method: The Time Kill Assay

The time-kill assay has been standardized and described in the M26-A document of CLSI [10]. As above, CuO NMs, or the negative or the positive control (190 µL) were added to 1.5 mL Eppendorf tubes, and then 10 µL of the bacteria inoculum added. The tubes were then vortexed and incubated at 37 °C in a shaking incubator for 2, 4, 8, 16, or 24 h. At each time point, 10 µL of each sample was diluted in distilled water via ten-fold serial dilutions, and then 10 µL of each dilution was plated on an MHA plate and incubated at 37 °C overnight. Viable bacteria were then counted (CFU).

The time-kill assay was also performed following preparation of the test substances in PBS using the method developed by Khan et al. (2008) [66]. The CuO NMs or positive or negative controls (190 µL, in PBS) were added to 1.5 mL Eppendorf tubes and then 10 µL of each bacterial suspension was added. The sample tubes were then vortexed and incubated at 37 °C in a shaking incubator for 2 h. Samples were then diluted in distilled water via ten-fold serial dilutions and 10 µL of the sample was plated onto the surface of MHA plate and incubated at 37 °C overnight before counting viable bacteria (CFU).

## 3. Viability Assays

### 3.1. Alamar Blue Assay

For the Alamar Blue assay, CuO NMs or the negative or positive controls (190 µL) were added to a 96 well plate. Next, bacteria (10 µL) were added to each well. To check for NM interference, wells that contained CuO NMs without bacteria were included. Each sample was incubated at 37 °C for 2 h. Next, 20 μL of 0.15 mg/mL resazurin dye (in MHB) was then added to each well and mixed thoroughly. The cells were incubated at 37 °C for 4 h. After incubation, fluorescence was measured at an excitation wavelength of 530 nm and emission wavelength of 590 nm using a microplate reader (FLUOstar Omega, BMG LABTECH, UK) [25].

### 3.2. Live/Dead Assay

In the live/dead assay, bacteria treated with different concentrations of CuO NMs or the negative or positive control as described for the Alamar Blue assay. The protocol was performed according to the instructions given by the kit manufacturer (ThermoFisher, LIVE/DEAD™BacLight™ Bacterial Viability Kit, L7012). To check for NM interference, CuO NMs were added to kit reagents without bacteria.

### 3.3. qPCR

To verify and distinguish between live and dead bacterial populations after NM exposure, qPCR was used. Each bacterial culture (1 mL) was added to Eppendorf tubes and treated with the LC_50_ concentration of CuO NMs in MHB (100 µg/mL for *E. coli* and *B. subtilis*, 200 µg/mL for *S. aureus* and *P. aeruginosa*) or the negative control. Samples were incubated at 37 °C for 2 h, before centrifugation (5000× *g* for 30 min) and re-suspension in 500 μL distilled water. A final concentration of 20 µM of PMA (Propidium monoazide, Avantor, 40013) in 20% DMSO was added to the sample and incubated for 5 min at RT in the dark. Samples were put on ice and subjected to a halogen light source (500 W) for 5 min with a 20 cm distance between the light source and samples [67]. After light exposure, bacteria were collected by centrifugation at 90005000× *g* for 10 min and stored at −20 °C until genomic DNA extraction. Genomic DNA extractions were performed using an extraction kit (PureLink™ Microbiome DNA Purification, A29790) according to the manufacturer’s instructions. DNA concentration and quality were assessed using UV absorbance in addition to the Fluorescence dye method by use of the QubitTM dsDNA HS Assay kit, Qubit3. Pure DNA samples comprise of a ratio between 1.7 and 2.0 when measured at the spectrophotometric relative absorbance ratio (260 nm/280 nm).

qPCR was performed using the Bio-Rad CFX Maestero PCR system (Bio-Rad Laboratories, Watford, UK). For *E. coli*, *S. aureus*, and *B. subtilis* 16S ribosomal RNA, primers were designed by using Sigma/OligoArchitecture. The *P. aeruginosa* primer sequence was taken from the published literature [67]. All primers were commercially synthesized by Applied Biosystem (UK). The list of selected primers is shown in Table 2. Amplification and detection were determined using PowerTrack SYBR Green Master Mix (Thermo, Cambridge, UK). DNA was amplified in 20 μL reaction volumes containing 0.3 μM of each primer. Briefly, the amplification profile was as follows: 95 °C for 2 min, 40 cycles at 95 °C for 15 s and 40 cycles 60 °C for 1 min. The RT PCR results were processed using the CFX Maestro software.

### 3.4. Statistical Analysis

All data are expressed as mean ± standard deviation (SD). The tests were performed in three separate experiments in triplicate samples. Statistical analyses were performed using Excel and GraphPad software. Statistical evaluation of data was performed by ANOVA and post hoc Tukey-test, enabling a pairwise comparison of the methods; significance was defined as *: *p* ≤ 0.05, **: *p* ≤ 0.01.

## 4. Results

### 4.1. CuO NM Characterization

The average hydrodynamic diameter of CuO NMs in water was 174.8 nm ± 4.2. The Polydispersity index (PDI) and Zeta potential were 0.28 ± 0.02 and −5.68 mV ± 3.94, respectively.

### 4.2. Well and Disc Diffusion

Using the well diffusion method, a clear zone of inhibition was observed for bacteria incubated with gentamicin (the positive control), whereas no inhibition zone was detected for bacteria exposed to MHB alone (the negative control) (Table 3). A concentration dependent increase in the size of the inhibition zone was observed for all bacteria exposed to CuO NMs. For *B. subtilis*, an inhibition zone was measured at concentrations ≥ 50 µg/mL, reaching a zone of inhibition of 4.0 mm at the highest concentration tested (200 µg/mL). In contrast, at a concentration of 50 µg/mL, no inhibition zone was found for *E. coli*, *S. aureus* or *P. aeruginosa*. However, inhibition zones could be measured at CuO NM concentrations of 100 and 200 µg/mL for *E. coli*, *S. aureus*, and at a concentration of 200 µg/mL only for *P. aeruginosa* (Table 3). Data obtained from the well diffusion assay was used to rank the sensitivity of bacteria to the antibacterial effects of CuO NMs (from most to least sensitive) as follows: *B. subtilis* ≥ *E. coli* > *S. aureus* > *P. aeruginosa*.

Using the disc diffusion method, we again observed a clear inhibition zone for all bacteria exposed to gentamicin (Table 3). For the negative control, there was no inhibition zone for any of the tested bacteria. Similarly, at CuO NM concentrations of 12.5, 25, and 50 µg/mL, there was no inhibition zone measured for any bacteria (Table 3). By contrast, an inhibition zone could be measured for *B. subtilis* and *E. coli* exposed to CuO NMs at concentrations of 100 and 200 µg/mL, with the largest inhibition zone observed for *B. subtilis*. Finally, an inhibition zone could only be measured at a concentration of 200 µg/mL for *S. aureus* and *P. aeruginosa*. Using the disc diffusion assay, the susceptibility of bacteria to the antibacterial effects of CuO NMs was ranked (from most to least sensitive) as follows: *B. subtilis* ≥ *E. coli* > *S. aureus* > *P. aeruginosa*.

For well and disc assays, only CuO NMs at a concentration of 200 µg/mL exhibited a statistically significant increase in the inhibition zone of bacterial culture for *B. subtilis*, *E. coli*, and *S. aureus* when compared to the control. No significant change in the inhibition zone was observed for *P. aeruginosa* compared to the control. The results also suggest that the well diffusion method was more sensitive than the disc diffusion method.

### 4.3. OD Measurement

**Figure 1:**
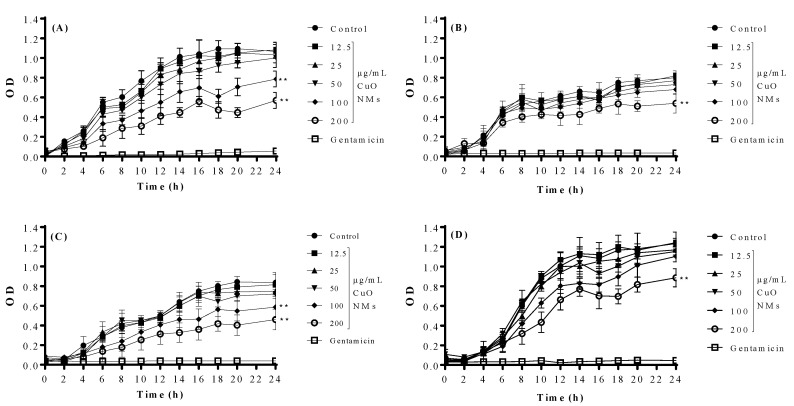
Impact of CuO NMs on bacterial growth (OD Measurement). Bacterial growth curves of (**A**) *E. coli* (**B**) *S. aureus* (**C**) *B. subtilis* and (**D**) *P. aeruginosa* following exposure to CuO NMs at concentrations ranging from 12.5 to 200 µg/mL for 24 h. Bacteria were also exposed to 100 µg/mL gentamicin (positive control) or incubated in MHB (negative control). OD measurements were made at 600 nm. These data represent the mean OD ± SD of three independent experiments done in triplicate. An ANOVA with a post hoc Tukey test was performed, with significant findings indicated by **: *p* ≤ 0.01, compared to the control.

For control bacteria, an increase in OD was observed over time which is indicative of bacterial growth (Figure 1). Exposure of bacteria to gentamicin prevented the increase in OD over time, with bacterial growth inhibited at all time points. CuO NMs caused a concentration dependent decrease in OD for all bacteria (Figure 1). The growth inhibition was most noticeable for *E. coli*, *B. subtilis*, and *P. aeruginosa*, with increasing growth inhibition observed for increasing NM concentrations and at longer time points. *S. aureus* was least susceptible to CuO NM toxicity (Figure 1). At 24 h post exposure, CuO NMs at a concentration of 100 μg/mL caused approximately 50% reduction in *E. coli* (Figure 1A) and *B. subtilis* (Figure 1C) growth when compared to the negative control. A statistically significant inhibition of growth was observed at CuO NM concentrations of 100 and 200 μg/mL for *E. coli* and *B. subtilis* when compared with control at 4, 8 and 24 h. However, only a concentration of 200 μg/mL CuO NMs caused a statistically significant inhibition of growth for *S. aureus* and *P. aeruginosa* over the period 8–24 h (Figure 1B,D). This suggests that *E. coli* and *B. subtilis* are more sensitive to CuO NM toxicity than *S. aureus* or *P. aeruginosa*.

### 4.4. Time-Kill Assay

**Figure 2:**
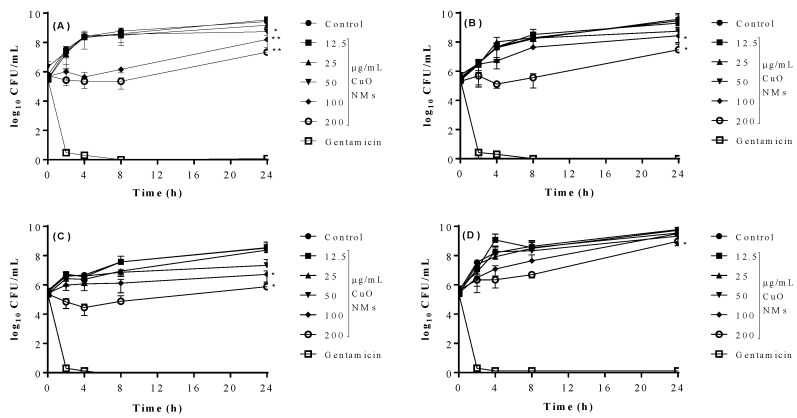
Impact of CuO NMs on bacterial growth (time-kill assay). Time-kill curves for (**A**) *E. coli*, (**B**) *S. aureus*, (**C**) *B. subtilis*, (**D**) *P. aeruginosa*. Bacteria were exposed to CuO NMs at concentrations ranging from 12.5 to 200 µg/mL (in MHB) for up to 24 h. Bacteria exposed to MHB (control) or 100 µg/mL gentamicin (positive control) were included. Viable cell counts were measured over time on MHA plates. Data expressed as mean CFU/mL ± SD of three independent experiments. An ANOVA with a post hoc Tukey test was performed, with significance indicated by *: *p* ≤ 0.05 and **: *p* ≤ 0.01, compared to the control.

For all tested bacteria, colony numbers dropped to zero after gentamicin exposure at all time points from 2 h, while the growth of bacteria in MHB (negative control) increased over time. For all bacteria, there was little difference between the growth of the bacteria when exposed to CuO NMs at concentrations of 12.5 and 25 µg/mL at all time points, when compared to the control (Figure 2). At the end of the experiment (24 h), CuO NMs caused a significant decrease in *E. coli* growth at concentrations of 50, 100, and 200 µg/mL (as indicated by a decrease in colony numbers) when compared to the control (Figure 2A). Significant reductions in the growth of *B. subtilis* and *S. aureus* were observed at CuO NM concentrations of 100 and 200 µg/mL, when compared to the control at 24 h. A significant reduction in *P. aeruginosa* growth was only observed at a CuO NM concentration of 200 µg/mL in comparison to control at 24 h (Figure 2D). For all bacterial strains, the greatest effect on growth was observed at a concentration of 200 µg/mL (Figure 2). This suggests that the growth inhibitory effect of CuO NMs can be ranked (from most to least sensitive) as follows: *E. coli* > *B. subtilis >* *S. aureus* > *P. aeruginosa*.

**Figure 3:**
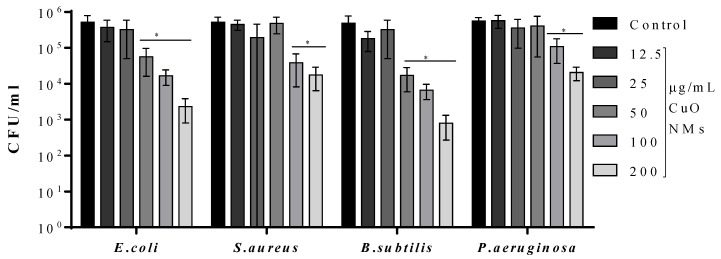
Impact of CuO NMs on bacterial growth at 2 h. *E. coli, S. aureus, B. subtilis*, and *P. aeruginosa* were exposed to CuO NMs at concentrations ranging from 12.5 to 200 µg/mL (in PBS). Viable cell counts were measured by culturing bacterial colonies on MHA plates. A negative control (bacteria in PBS) and positive control (100 µg/mL gentamicin) were included. Data is expressed as mean CFU/mL ± SD of three independent experiments done in triplicate. An ANOVA with a post hoc Tukey test was performed, with significance indicated by *: *p* ≤ 0.05, compared to the control.

The time-kill assay was also performed using PBS instead of MHB, with a 2 h incubation time to confirm the antibacterial activity of CuO NMs. Bacteria exposed to 100 µg/mL of gentamicin exhibited 100% killing. Compared to the control, it was observed that CuO NMs caused a significant reduction in the number of viable *E. coli* and *B. subtilis* at concentrations of 50, 100 and 200 µg/mL. Moreover, at concentrations of 100 and 200 µg/mL, CuO NMs caused a significant reduction in the number of viable *S. aureus* and *P. aeruginosa* (Figure 3). Based on the results of this assay, the ranking of effect (from most to least sensitive) was: *B. subtilis* ≥ *E. coli* > *S. aureus* = *P. aeruginosa*.

### 4.5. Viability Assays

**Figure 4:**
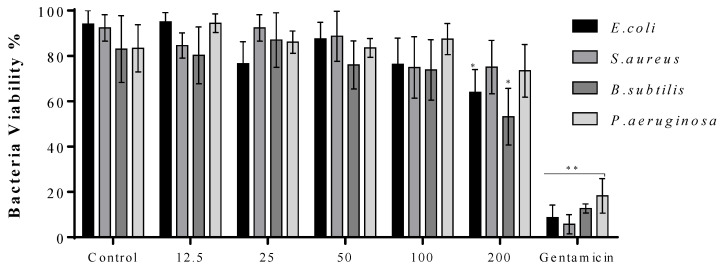
The impact of CuO NMs on the viability of bacteria (Alamar Blue assay). *E. coli, S. aureus, B. subtilis*, and *P. aeruginosa* were exposed to CuO NMs (12.5–200 µg/mL) or the positive (100 µg/mL gentamicin) or negative (MHB) controls at 37 °C for 2 h and cell viability assessed using the Alamar Blue Assay. Data expressed as mean bacteria viability (% of negative control) ± SD of three independent experiments done in triplicate. An ANOVA with a post hoc Tukey test was performed, with significance represented by *: *p* ≤ 0.05, and **: *p* ≤ 0.01, compared to the control.

The viability of bacteria when exposed to various concentrations of CuO NMs for 2 h was determined using the Alamar Blue assay. CuO NMs caused a significant decrease in the viability of *E. coli* and *B. subtilis* only at the highest concentration tested (200 µg/mL) (Figure 4) with no significant reduction in viability observed for *S. aureus* or *P. aeruginosa* at any concentration.

**Figure 5:**
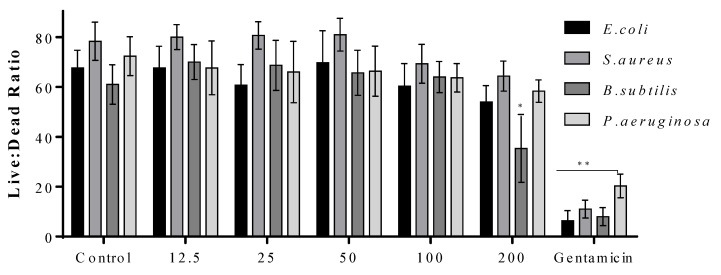
The impact of CuO NMs on bacteria viability using the Live/dead assay. *E coli, S. aureus, B. subtilis*, and *P. aeruginosa* were exposed to CuO NMs (12.5–200 µg/mL), the positive (100 µg/mL gentamicin) or negative (MHB) control at 37 °C for 2 h and the live/dead assay performed. Data expressed as mean live:dead ratio ± SD of three independent experiments. An ANOVA with a post hoc Tukey test was performed, with significance represented by *: *p* ≤ 0.05 and **: *p* ≤ 0.01 compared to the control.

The live/dead assay was also performed to quantify the impact of CuO NMs on bacterial viability at 2 h. It was found that when treated with all concentrations CuO NMs, *E. coli*, *S. aureus*, and *P. aeruginosa* showed no significant difference in the ratio of live:dead cells, compared to control (Figure 5). The only significant inhibition in cell viability was observed for *B. subtilis* exposed to CuO NMs at a concentration of 200 µg/mL in comparison with the control (Figure 5).

**Figure 6:**
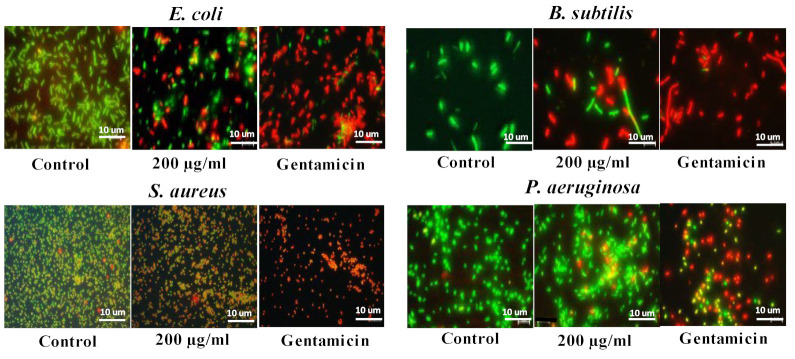
Imaging of live and dead cells following CuO NM exposure. Representative microscopic images of bacteria exposed to CuO NMs, MHB (negative control) and gentamicin (positive control). Bacteria (*E. coli*, *S. aureus*, *B. subtilis*, and *P. aeruginosa*) were exposed to CuO NMs at a concentration of 200 µg/mL for 2 h and stained using the live/dead stain to visualise dead (red) and viable (green) cells. Cells were imaged at 100× magnification using a Leica DM IRBE CLSM. Scale bar is 10 μm.

Cells were also imaged using fluorescent microscopy after staining with the live/dead stain. Live cells with an intact membrane appear green, and dead cells appear red (Figure 6). The majority of control (untreated) cells for all bacteria were viable, with very few dead cells. Nearly all bacteria exposed to gentamicin were dead for all bacterial strains. Following exposure to CuO NMs, a concentration-dependent decrease in cell viability was observed (Figure 6). At low CuO NMs concentrations (50 µg/mL), no cytotoxic effects were observed for all of the bacteria, and most of the cells were viable (Figure S2). In contrast, at a concentration of 100 µg/mL, the number of dead cells increased for all bacteria following CuO NM exposure (Figure S2). At a concentration of 200 μg/mL CuO NMs, the highest level of cell death was observed, with the greatest effect observed for *E. coli* and *B. subtilis* (Figure 6).

### 4.6. qPCR

**Figure 7:**
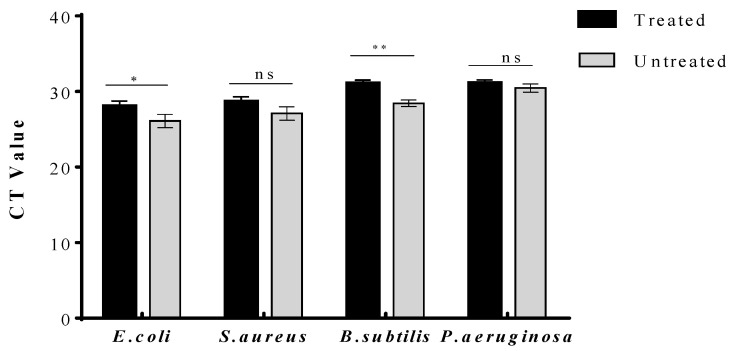
Impact of CuO NMs on bacterial growth: the viability qPCR assay. Bacteria were treated with CuO NMs (100 µg/mL for *E. coli* and *B. subtilis*, 200 µg/mL for *S. aureus* and *P. aeruginosa*) or MHB (untreated, or negative control) and incubated for 2 h. PMA was added and DNA extracted, and qPCR was performed using the Bio-Rad CFX Maestero PCR system. Data expressed as mean CT value ± SD of three independent experiments. An ANOVA with a post hoc Tukey test was performed and statistical significance indicated by *: *p* ≤ 0.05, **: *p* ≤ 0.01, and ns: non-significant compared to the control.

To distinguish live and dead bacteria using qPCR, a viability qPCR assay was performed in the presence of PMA. When the initial concentration of the target template (16 S ribosomal DNA) is high, the CT will be reached at an earlier amplification cycle. For *E. coli* and *B. subtilis*, a significant increase in the CT value was observed following exposure to CuO NMs at a concentration of 100 µg/mL, indicating the antibacterial activity of CuO NMs against both bacteria (Figure 7). No significant change in the CT value was observed for *S. aureus* or *P. aeruginosa*, suggesting that CuO NM treatment had no significant effect on cell viability.

## 5. Discussion

Numerous approaches have been used to assess the antibacterial effect of NMs in vitro. To date, plate counts and well/disc diffusion assays have been most commonly applied to detect the antibacterial properties of NMs. However, alternative methods such as the use of viability assays, measurement of OD and PCR could be adopted more widely to make testing more rapid and with higher throughput. So far, the various antimicrobial methods available have not been systematically assessed for their suitability and sensitivity for NM testing. Here, we assessed the antibacterial activity of CuO NMs to strains of four species of bacteria (*B. subtilis*, *E. coli*, *S. aureus* and *P. aeruginosa*) using a battery of tests. All assays confirmed that CuO NMs exhibited antibacterial properties. The sensitivity of the different bacterial strains tested to CuO NM toxicity was ranked (from most to least sensitive) as follows: *B. subtilis* ≥ *E. coli* > *S. aureus* > *P. aeruginosa*. Interestingly, the assays varied with respect to their sensitivity, cost, time, technical difficulty and requirement for access to special equipment. The prioritisation of specific assays is required to streamline the assessment of the antibacterial properties of NMs. We therefore propose that our findings can inform the selection of assays used in the future to assess the antibacterial properties of NMs.

### 5.1. Assay Sensitivity

We demonstrated that the range of assays we used varied in their sensitivity to detect the antimicrobial effects of CuO NMs. The time-kill assay was the most sensitive approach tested whereas the viability assays were the least sensitive. Investigation of the antibacterial properties of NMs has been most commonly assessed by performing plate counts at one time point or several time points (time-kill assay) in the published literature. While plate counts are a sensitive approach, they are time consuming and more labour intensive to perform than other approaches.

Well and disc diffusion methods have also been commonly used to assess the antibacterial properties of NMs in published studies. These assays are relatively quick and easy to perform but the diffusion capability of NMs should be considered as a limitation of this method [10]. In this study, the well and disc diffusion methods lacked sensitivity, with significant effects observed only at the highest concentration of CuO NMs tested. This is likely to be due to the limited movement of CuO NMs from the discs which influences their ability to physically interact with the bacterial cells [32]. Accordingly, the small inhibition zones observed is attributed to copper ion release from the NMs which could penetrate into the culture media [32]. Despite the limitations of this assay, the results we obtained did however allow a relative ranking of the bacterial strain sensitivity that was comparable to the time-kill (plate count) method.

Measurement of OD over a course of time has been less commonly used in the published literature to assess the impact of NMs on bacterial growth. We found that this approach allowed for a rapid, low cost and high throughput assessment of the antibacterial properties of NMs and would therefore recommend its more widespread use. However, NMs can contribute to OD readings and thereby interfere with the assessment of bacterial growth; therefore, appropriate controls (e.g., measurement of OD in the absence of bacteria) are essential to correctly interpret the data. Interestingly, published studies often neglect to mention whether NM interference was assessed when assessing the antibacterial activity of NMs, but we recommend that the approach used to assess for NM interference should be clearly stipulated in the methodology to allow appropriate interpretation of the results.

Even though viability assays are not extensively used currently to assess the antibacterial activity of NMs, they are quicker to perform and have higher throughput than more traditional approaches. The live/dead assay, Alamar Blue assay or assessment of ATP production have been used to assess the antibacterial properties of substances, including NMs [27,36,68,69]. Furthermore, He et al. (2016) demonstrated the antibacterial activity of MgO NMs against *Escherichia coli*, *Campylobacter jejuni*, and *Salmonella enterica* using the Alamar Blue assay [25]. However, in our study, the Alamar Blue and the live/dead assays lacked sensitivity, identifying effects only for *B. subtilis* treated with the highest concentration of CuO NMs tested. It is established that the dyes used in our study are sensitive to interference by a variety of NMs [70,71], and so as discussed above, it is essential to assess NM interference with these assays as they are often colorimetric/fluorescent and the optical properties of NMs may interfere with their performance. For example, NMs may affect the absorbance, fluorescence, or luminescence read-outs. NMs may also interfere directly with the assay by interacting with the substrate and/or product, or by catalysing the conversion of the substrate to its product [72,73].

Use of PCR is increasing in popularity when assessing the antibacterial properties of substances, including NMs [74]. However, we found that PCR did not provide a sensitive assessment of the antibacterial properties of CuO NMs. It is possible that NMs might interfere with qPCR amplification as it is established that NMs can bind to DNA [75,76]. Moreover, the cost and requirement to access specialised equipment for PCR may be prohibitive to some researchers. However, further optimisation of the protocol used could improve the sensitivity of this assay to potentially make testing more rapid, and with higher throughput.

Whilst highly-standardised methods are available for antimicrobial susceptibility testing (minimum inhibitory concentration (MIC), minimum bactericidal concentration (MBC) and well/disc diffusion assays) and other assays which monitor growth, inhibition and/or viability (plate counts, OD, dose-response and time-kill) are widely applied to investigate antibacterial activity, these protocols may need to be modified to be suitable to assess NMs. For example, a recent study indicated that substantial dilution of NM-treated bacteria into fresh growth medium guarantees minimal optical interference and toxic influence from NMs in the exposure mixture when measuring optical density [77]. In addition, standard methods for the viability assays and PCR need to be developed to ensure a harmonised approach is used internationally.

Based on the findings of our study, we recommend that a combination of assays is employed to confirm the antibacterial activity of NMs. More specifically, the plate count (time-kill) method should be prioritised and developed further to make it higher in throughput, because it has the advantage that it lacks interference by NMs [18]. We also suggest that measurement of OD over time should be prioritised as it is a high throughput approach, although it is essential to assess NM interference in parallel. These assays were relatively sensitive, allowing detection of effects at lower NM concentrations for a wider number of bacterial strains. The suitability of using well/disc diffusion assays to assess NM toxicity requires further exploration. The well/disc diffusion assays, viability assays and PCR were less sensitive and have the potential for NM interference and so it is recommended that their use is not prioritised.

### 5.2. Sensitivity of Bacterial Strains

We tested two Gram-negative (*E. coli*, *P. aeruginosa*) and two Gram-positive (*B. subtilis, S. aureus*) strains of bacteria in our study. Whilst CuO NMs exhibited antibacterial effects to all strains of bacteria tested in our study, the bacteria varied with respect to their sensitivity to CuO NM toxicity, with *B. subtilis* and *E. coli* observed to be the most sensitive strains. Whilst we did not observe that Gram-negative bacteria were more sensitive to the toxicity of NMs than Gram-positive strains, other studies have made this observation. For example, silver NMs exhibited greater antibacterial activity against *E. coli* than *S. aureus*, which was attributed, in part, due to differences in the cell wall structure of these bacteria [78]. More specifically, the cell envelope of Gram-negative bacteria is composed of an outer membrane containing lipopolysaccharides, which surrounds a thin layer of peptidoglycans and an inner cytoplasmic membrane [78]. Furthermore, binding of NMs to anionic surface domains of Gram-negative bacteria may enhance their toxicity [79]. The thick protective peptidoglycan layer (with covalently attached teichoic and teichuronic acids) of Gram-positive bacteria may limit the uptake of NMs or their released ions to provide protection from NM toxicity [80]. Interestingly, several studies have shown the contrary—that Gram positive bacteria are more sensitive to the toxicity of NMs than Gram-negative bacteria. For example, Azam et al. (2012b) [50] examined the antibacterial efficacy of CuO, ZnO, and Fe_2_O_3_ NMs to *E. coli*, *P. aeruginosa, B. subtilis* and *S. aureus* using well diffusion and broth dilution (plate count) methods, and showed that *B. subtilis* was most sensitive. *B. subtilis* was also more sensitive to CeO2 NM toxicity than Shewanellaoneidensis (Gram-negative) which was found to be resistant to NM activity [81]. The apparent discrepancies in the sensitivity of different bacteria in the published literature is likely due to differences in the experimental design used in different studies (e.g., NM concentrations tested, time points, bacterial strains, and approach used to assess toxicity) as well as to physico-chemical properties of the NMs tested (e.g., size, solubility, shape, surface area, etc) and, significantly, to substantial genotypic and phenotypic differences among the bacteria selected for assays. Interestingly, many existing nanotoxicology studies have focused on the same species of bacteria (e.g., *E. coli*, *P. aeruginosa* and *S. aureus*). Therefore, the use of more diverse strains in the future would help identify how a wider variety of bacteria vary in their sensitivity to NM toxicity.

## 6. Conclusions

In the current study, numerous tests were applied to test the antibacterial activity of CuO NMs. The results demonstrated that the time-kill and OD assays were the most sensitive methods and should be prioritised in the future. The assessment of the sensitivity of different assays provided here will be useful in informing others in the design of studies which investigate the antibacterial efficacy of NMs in the future.

## Figures and Tables

**Table 1:** Comparison of commonly used methods for evaluating the antibacterial activity of NMs.

Method	Advantages	Disadvantages
Agar diffusion	▪ Simple, flexible, low-cost, straightforward, easy to interpret results and does not require any special equipment [31].	▪ Unsuitable for slow growing and anaerobic bacteria; lack of automation of the test. ▪ Dependent on the size of NMs. Moreover, the *w*/*v* ratio of agar might affect particle dispersal and low diffusivity for NMs [32]. ▪ Provides only qualitative results in 18–24 h [33,34]. ▪ False negative results may arise from the failure of nonpolar compounds to diffuse, or when testing cationic compounds as these can adsorb onto the disc paper [35]. ▪ This method cannot differentiate between bacteriostatic and bactericidal effects [36].
Time-kill	▪ High accuracy [37], low-cost, simple to use and requires minimum training to perform.	▪ Time consuming, large amounts of materials required [37]. ▪ Issues arise when viable but non-culturable bacteria are present as they can no longer grow on standard media [38]. ▪ Determines CFU count but not entire bacterial cell culture or size of CFU [37].
Optical density	▪ Reproducible, not technically challenging, can be performed with small amounts of antimicrobials, and quantitative data is generated [34,36]. ▪ Non-destructive [39]. ▪ Bactericidal and bacteriostatic effects can be distinguished [35]. ▪ Amenable to high throughput testing.	▪ Spectrophotometer required [37]. ▪ Numerous steps in sample preparation which increases the probability of errors. ▪ Not suitable for bacteria concentrations less than 10^5^ CFU/mL [39] ▪ Unreliable when bacteria are cultivated with a substance (e.g., NMs) that could interfere with absorbance [39]. Therefore difficult to test coloured compounds. ▪ Difficult to test hydrophobic compounds.
Alamar Blue	▪ Few wash steps involved, and follow-up assays can be performed on same cells as assay is not cytotoxic [40]. ▪ High throughput.	▪ Conversion to resorufin dependant on enzymatic conversion [40].
LIVE/DEAD BacLight viability	▪ Rapid assessment of antibacterial effects [26]. ▪ Microscopy can be used to visualize the impact of the test substance on bacterial cell viability [37].	▪ Variable results and cannot be used to verify species of bacteria [26]. ▪ Expensive reagents, microscope or fluorescent plate reader needed [37]. ▪ Time consuming [41].
qPCR	▪ Safe, effective, and reliable [42]. ▪ All bacteria have at least one copy of 16 S rDNA for which many sequences are known, therefore it can be relatively straightforward to implement targeted assays.	▪ Identifying which genes to analyse is critical to reduce the chance of detecting false resistance [42].

**Table 2:** Primers for sequence amplification and detection (qPCR).

Target	Primer	Sequence
*E. coli*	Primer (F)	5′-TAATACCTTTGCTCATTG-3′
	Primer (R)	5′-CCAGTAATTCCGATTAAC– 3′
*P. aeruginosa*	Primer (F)	5′-TCC AAG TTT AAG GTG GTA GGC TG-3′
	Primer (R)	5′-CTT TTC TTG GAA GCA TGG CAT C-3′
*S. aureus*	Primer (F)	5′-CGTGCTACAATGGACAATA-3′
	Primer (R)	5′-CCGAACTGAGAACAACTT-3′
*B. subtilis*	Primer (F)	5′-GCTACAATGGACAGAACAA-3′
	Primer (R)	5′-ATCCGAACTGAGAACAGA-3′

**Table 3:** Investigation of the antibacterial properties of CuO NMs using well and disc agar diffusion assays. The bacterial suspension was seeded on the surface of MHA in Petri dishes, with four wells/discs in each dish. The wells/discs were loaded with selected concentrations of CuO NMs or the negative (MHB alone) or positive (100 µg/mL gentamicin) control then incubated at 37 °C for 18 h. The zone of inhibition was measured (in mm), with well/disc diameters (6 mm) subtracted. R signifis that bacteria are resistant (no measurable inhibition zone). Data expressed as mean Inhibition Zone (mm) ± SD of three independent experiments done in triplicate. An ANOVA with a post hoc Tukey test was performed, with significance indicated by *: *p* ≤ 0.05, and **: *p* ≤ 0.01 compared to the control.

		*Inhibition Zone (mm)*			
Method	Treatment (µg/mL)	*E. coli*	*S. aureus*	*B. subtilis*	*P. aeruginosa*
Well Diffusion	CuO NMs 12.5	R	R	R	R
	CuO NMs 25	R	R	R	R
	CuO NMs 50	R	R	0.5 ± 0.5	R
	CuO NMs 100	1.5 ± 1.0	1.0 ± 1.0	2.0 ± 1.8	R
	CuO NMs 200	3.0 ± 1.0 **	2.5 ± 0.5 **	4.0 ± 0.5 **	1.5 ± 1.0
	Gentamicin 100	20.0 ± 1.8 **	18.0 ± 2.0 **	24.0 ± 4.0 **	12.0 ± 4.0 **
	MHB alone (control)	0	0	0	0
Disc Diffusion	CuO NMs 12.5	R	R	R	R
	CuO NMs 25	R	R	R	R
	CuO NMs 50	R	R	R	R
	CuO NMs 100	1.5 ± 1.3	R	1.5 ± 1.0	R
	CuO NMs 200	2.0 ± 1.0 *	1.6 ± 0.9 *	3.1 ± 1.7 **	1.0 ± 0.7
	Gentamicin 100	16.0 ± 3.0 **	12.0 ± 3.6 **	17.5 ± 3.1 **	7.0 ± 2.0 **
	MHB alone (control)	0	0	0	0

## Data Availability

The data presented in this study are available on request from the corresponding or lead author.

## Supplementary Materials

The following supporting information can be downloaded at: https://www.mdpi.com/article/10.3390/jfb13040255/s1, Figure S1: Assessment of antibacterial activity using well (left hand side) and disc (right hand side) diffusion methods. *E. coli* (A), *S. aureus* (B), *B. subtilis* (C), and *P. aeruginosa* (D) were exposed to CuO NMs at concentrations of 200 µg/mL (a); 100 µg/mL (b); or 50 µg/mL (c); Mueller Hinton Broth– the negative control (d); CuO NMs at concentrations of 25 µg/mL (e); 12.5 µg/mL (f); or Gentamicin—the positive control at a concentration of 100 µg/mL (g). Images of the inhibition zone were taken at 18 h post exposure; Figure S2: The impact of CuO NMs on cell viability: the live/dead assay. Bacteria (*E. coli, S. aureus, B. subtilis*, and *P. aeruginosa*) were exposed to CuO NMs at a concentration of 50 or 100 µg/mL for 2 h and stained using the live/dead stain (ThermoFisher, LIVE/DEAD™BacLight™ Kit, L7012) to visualise dead (red) and viable (green) cells. Cells were imaged at 100× magnification using a Leica DM IRBE CLSM. Scale bar is 10 µm. Representative microscopic images are presented.
