# Supplementary material for: Comparison of In Vitro Approaches to Assess the Antibacterial Effects of Nanomaterials
Source: J Funct Biomater. 2022 Nov 19;13(4):255. doi: 10.3390/jfb13040255 (PMC9703965; doi:10.3390/jfb13040255)
Supplement: Supplementary file 1 [file jfb-13-00255-s001.zip › jfb-1999960-supplementary.pdf]

Supplementary Materials

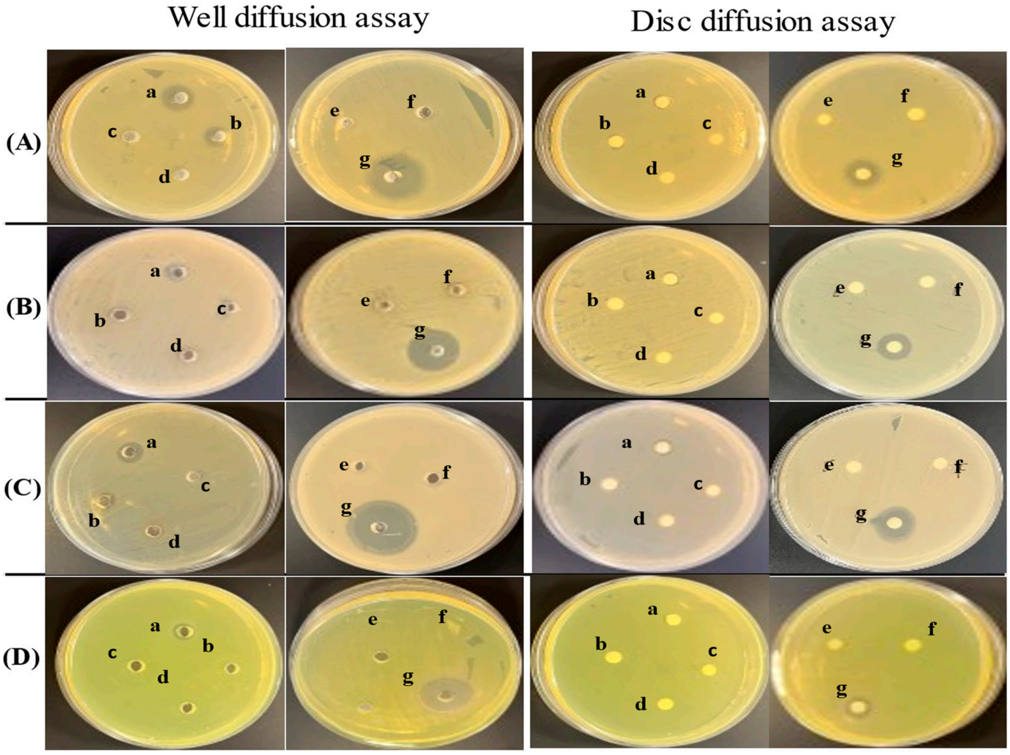

Figure S1. Assessment of antibacterial activity using well (left hand side) and disc (right hand side) diffusion methods. *E. coli* (A), *S. aureus* (B), *B. subtilis* (C), and *P. aeruginosa* (D) were exposed to CuO NMs at concentrations of 200 μg/mL (a); 100 μg/mL (b); or 50 μg/mL (c); Mueller Hinton Broth– the negative control (d); CuO NMs at concentrations of 25 μg/mL (e); 12.5 μg/mL (f); or Gentamicin—the positive control at a concentration of 100 μg/mL (g). Images of the inhibition zone were taken at 18 h post exposure.

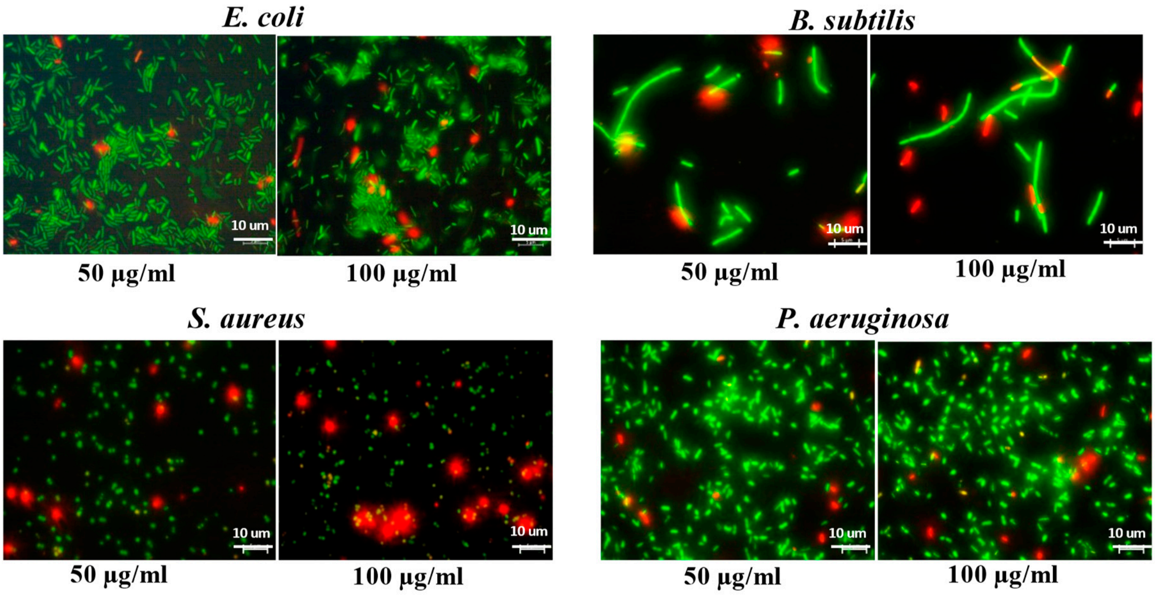

Figure S2. The impact of CuO NMs on cell viability: the live/dead assay. Bacteria (*E. coli, S. aureus, B. subtilis,* and *P. aeruginosa*) were exposed to CuO NMs at a concentration of 50 or 100 µg/mL for 2 h and stained using the live/dead stain (ThermoFisher, LIVE/DEAD™BacLight™ Kit, L7012) to visualise dead (red) and viable (green) cells. Cells were imaged at 100 X magnification using a Leica DM IRBE CLSM. Scale bar is 10µm. Representative microscopic images are presented.
